# Describing Supportive Care Programming Access and Comfort Gathering through the COVID-19 Pandemic: An Observational Mixed Methods Study with Adults Affected by Cancer

**DOI:** 10.3390/curroncol30030198

**Published:** 2023-02-22

**Authors:** Amanda Wurz, Anna Janzen, Kelsey Ellis, Iris Lesser, Nafeel Arshad

**Affiliations:** 1 School of Kinesiology, University of the Fraser Valley, Chilliwack, BC V2R 0N3, Canada; 2 Faculty of Kinesiology, University of Calgary, Calgary, AB T2N 1N4, Canada; 3 Faculty of Medicine, University of Toronto, Toronto, ON M5S 1A8, Canada

**Keywords:** oncology, well-being, qualitative, perspectives, telehealth

## Abstract

Supportive care programming helps many adults affected by cancer manage concerns related to their disease. Public health restrictions imposed by the COVID-19 pandemic have undoubtedly changed the nature of supportive care programming delivery. Yet, access to supportive care programming and comfort gathering through the pandemic are unknown. As a first step towards informing ongoing supportive care programming for adults affected by cancer, this observational, mixed methods study described supportive care programming access through the COVID-19 pandemic and comfort returning to in-person supportive care programming as restrictions eased. Adults affected by cancer (*n* = 113; mean age = 61.9 ± 12.7 years; 68% female) completed an online survey, and descriptive statistics were computed. A purposeful sample of survey participants (*n* = 12; mean age = 58.0 ± 14.5 years; 58% female) was subsequently recruited to complete semi-structured interviews. Interviews were analyzed using reflexive thematic analysis. Less than half (41.6%) of the survey sample reported accessing supportive care programming during the pandemic, and of those who had accessed supportive care programming, most (65.6%) perceived similar or greater access than pre-pandemic. During interviews, participants described the ways online delivery enhanced their access and reduced barriers to supportive care programming. However, physical activity programming was described as challenging to navigate online. With restrictions easing, most of the survey sample (56.6%) reported being apprehensive about returning to in-person supportive care programming and identified the protocols that would make them feel safe to gather. During interviews, participants recounted struggling to balance their need for social connection with their health and safety. This study provides evidence to inform supportive care programming for adults affected by cancer through the COVID-19 pandemic. Findings suggest online delivery can enhance access to some types of supportive care programming for some adults affected by cancer, and that efforts are needed to ensure all adults affected by cancer feel comfortable gathering in-person.

## 1. Introduction

It is estimated that two in five Canadians will be diagnosed with cancer in their lifetime [1] and that currently over 1.5 million adults are affected by cancer (i.e., living with and beyond cancer) in Canada [2]. Though survival rates continue to improve [1,3], cancer is associated with poor health, secondary cancers, and morbidity [4,5]. Through the COVID-19 pandemic, adults affected by cancer have experienced additional adverse health consequences [6]. Recent reports suggest heightened loneliness, stress, anxiety, and depression, and decreased quality of life, physical activity, and physical function [7,8,9,10]. This is perhaps unsurprising as adults affected by cancer have been tasked with managing their disease and its sequelae amidst public health restrictions and the re-allocation of healthcare resources that in many cases limited access to treatment and supportive care programming [11]. 

Supportive care programming (i.e., resources that help adults affected by cancer manage concerns related to their disease) is viewed as an essential adjunct to cancer care and may include psycho-oncology (e.g., support groups) and integrative medicine (e.g., physical activity or yoga programs) [12]. Supportive care programming has commonly been delivered in-person (face-to-face) in clinic- or community-based settings. However, at the onset of the COVID-19 pandemic, public health mandates were put in place to manage viral spread and in-person delivery was restricted [13]. These changes undoubtedly altered the way supportive care programming was delivered to adults affected by cancer. Yet, access to supportive care programming and experiences with changed supportive care programming among adults affected by cancer has been under explored. 

Further, as the pandemic progresses and restrictions lift, it is unclear what impact returning to in-person supportive care programming may have on perceptions of and comfort gathering among adults affected by cancer. For example, gathering in-person may enhance the likelihood of being exposed to and contracting COVID-19, and adults affected by cancer are at risk of severe COVID-19-related complications if infected (e.g., delayed treatment, unnecessary hospitalization; [14,15,16]). However, COVID-19 transmission has been shown to vary across environments, with lower infection risk associated with gathering in-person outdoors (as compared to indoors; [17]). Understanding perceptions of and comfort gathering in different environments among adults affected by cancer may help inform supportive care programming for this cohort through the pandemic. 

The COVID-19 pandemic and associated public health restrictions have undoubtedly impacted health and cancer care. Yet, access to supportive care programming and comfort gathering in this cohort remains unknown. As a first step towards informing supportive care programming for adults affected by cancer through the COVID-19 pandemic, this study described perceptions of supportive care programming access through the COVID-19 pandemic and comfort returning to in-person supportive care programming (indoors and outdoors) as restrictions eased in Canada. 

## 2. Methods

Gathering quantitative and qualitative data was deemed essential to advance understanding beyond what could be possible through one methodology alone. Thus, an observational, sequential explanatory mixed methods study was conducted. A pragmatic approach was adopted [18] and data were collected in two steps: (i) an online survey and (ii) semi-structured interviews. 

### 2.1. Recruitment and Participants 

Potential participants were recruited through the study team’s contacts (researchers across Canada), cancer support organizations, social media (paid advertisement on Facebook), and snowball sampling. To participate in this study, individuals had to affirm they were: (i) an adult ≥ 19 years of age, (ii) diagnosed with cancer ≥ 18 years of age, (iii) at any stage along the cancer trajectory (i.e., pre-treatment, on-treatment, completed treatment, palliative care), and (iv) residing in Canada. 

### 2.2. Procedures

#### 2.2.1. Online Survey

The online survey took place between December 2021 and February 2022, during the fifth wave of the COVID-19 pandemic in Canada and when the highly contagious Omicron caused a rapid increase in cases and public health restrictions were heightened. After completing online informed consent, participants were assigned an identification number and were given access to a series of questionnaires in SurveyMonkey. First, a socio-demographic and medical survey (e.g., age, sex, cancer history), the 10-item Perceived Stress Scale (PSS; [19]) and 8-item UCLA Loneliness Scale (ULS-8; [20]) were completed to describe the sample. Next, two researcher-generated questionnaires (see Supplementary File S1) were administered to explore supportive care programming access and comfort gathering in-person. 

#### 2.2.2. Semi-Structured Interviews

In March 2022, concurrent with when there was a decline in the number of COVID-19 cases and an easing of public health restrictions, the survey was closed. The study team explored all participant survey responses and purposefully selected a subset of participants to complete 1:1, online interviews following a semi-structured interview guide. Participants were selected based on variables of interest (i.e., socio-demographic characteristics and levels of stress and loneliness) and responses to the researcher-generated questionnaires. The selected subset of survey participants received an email from the study team inviting them to take part in an interview. Interviews were conducted over Zoom by one of two undergraduate students (N.A., A.J.) who identify as male and female, respectively. N.A. and A.J. underwent extensive training (in addition to their undergraduate research methods course work) prior to conducting interviews. Specifically, over several weeks, N.A. and A.J. completed a series of readings and reflection prompts and attended workshops delivered by the first author (A.W.) covering the methodological approach, building rapport, and probing. They also practiced conducting qualitative interviews with one another, their peers, and other members of the first author’s lab and practiced reviewing and analyzing qualitative data (using the same approach) from other studies. Questions asked during the interview were designed to further explore participants’ experiences with supportive care programming during COVID-19 and perceptions of comfort gathering in-person. Interviews lasted on average 51.3 ± 24.6 min.

### 2.3. Sample Size

For the online survey, an a priori target of 100 participants was set. For the semi-structured interviews, a purposeful subset of participants was recruited until a diverse range of perspectives was acquired and the widely used criterion of saturation was reached [21]. Throughout data collection, saturation was monitored, and sampling was discontinued when no new information was heard during the interviews.

### 2.4. Data Analysis

Data from the closed-ended questionnaires were analyzed using IBM SPSS (Version 27). Of the 160 who completed online informed consent, 47 were excluded from the analyses due to missing data (i.e., no response on questionnaires). Descriptive statistics (e.g., means, standard deviations) were computed for individuals who completed the survey. 

Semi-structured interviews were transcribed verbatim, managed in NVivo 12, and analyzed via reflexive thematic analysis [22]. Three study team members (A.W., N.A., A.J.) familiarized themselves with the data. Next, two study team members (N.A., A.J.) used an inductive approach to independently generate codes. Codes were reviewed and similar codes were grouped together to create themes and subthemes. During this process, A.W., N.A., and A.J. met several times and exchanged several drafts comparing their interpretations of the data. Through this iterative process, a theme table was drafted, including theme and subtheme labels, descriptions, and representative quotes. This table was then shared with two study team members (K.E., I.L.) who critically reviewed and commented on participants’ reflections and researcher interpretations. Following this, a final theme table was drafted. Upon completing interview analysis, all quantitative and qualitative data were reviewed together by the study team to better understand participants’ experiences with supportive care programming through the pandemic and comfort gathering in-person. 

## 3. Results

The quantitative and qualitative findings from this study are presented together across three sections. The first section, **The Context**, describes key characteristics of the survey and interview samples, offers deeper insight into participants’ mental health through the pandemic, and describes participants’ cancer and supportive care experiences as a means of situating the main findings to follow. The second section, **Main Findings**, describes participants supportive care programming use and comfort gathering through the pandemic. This is followed by a third section, **Practical Suggestions for Moving Forward in the Context of COVID-19**, which describes safety protocols that were endorsed by participants. Of note, Table 1 provides summarized survey and interview participant characteristics, Table 2 provides additional anonymized quotes to support each section, and Table 3 provides the descriptive details of interview participants who are quoted herein. In the quotes to follow, pseudonyms have been used to protect anonymity, identifying details have been removed, and […] signifies text that was removed for clarity.

### 3.1. The Context

#### 3.1.1. Participants

As seen in Table 1, the analytic survey sample was comprised of 113 adults affected by cancer who were on average 61.9 (SD = 12.7; range = 21–84) years of age. Most self-identified their biological sex as female (*n* = 77; 68.1%) and reported being of British (*n* = 65; 57.5%) or Western European descent (*n* = 33; 29.2%) and living in an urban setting (*n* = 75; 66.4%). Participants reported having been diagnosed with cancer between 1991 and 2021 (Mean time since diagnosis = 56.8, SD = 69.8 months) and were at varying stages along the cancer trajectory. Genitourinary (*n* = 30; 26.5%), breast (*n* = 30; 26.5%), and blood (*n* = 22; 19.5%) cancers were among the most common cancers diagnosed. Most participants self-reported being immunocompromised (*n* = 68, 60.2%), and 48 (42.5%) had been told by their physician that they were immunocompromised. 

Twenty-six participants were contacted to complete interviews. Of these, 1 was unreachable (i.e., undeliverable email), 17 responded, and 12 were available and agreed to schedule interviews. Visual inspection of the data suggests unreachable and declining participants (*n* = 13) were similar in terms of key demographic (i.e., age, ethnic background) and medical (i.e., cancer diagnosis, time since diagnosis) characteristics to those who agreed to participate. The interview sample was comprised of 12 participants (*n* = 7; 58.3% female) who were on average 58.0 (SD = 14.5; range = 35.0–77.0) years of age and self-reported being of varied ethnic backgrounds. Participants had been diagnosed with cancer between 2004 and 2021 (mean time since diagnosis = 52.8, SD = 62.9 months). 

#### 3.1.2. Mental Health through COVID-19

Overall, survey and interview participants reported moderate levels of stress (mean survey participants = 16.9, SD = 8.2; mean interview participants = 22.4, SD = 12.0). Survey participants also reported normal to moderate levels of loneliness (mean survey participants = 17.5, SD = 5.4), whereas interview participants reported moderate to high levels of loneliness (mean interview participants = 21.4, SD = 6.6). During interviews, participants elaborated on their mental health and the impact the COVID-19 pandemic had on their well-being.

Most participants described feelings of overwhelming stress, distress and depressive symptoms, anxiety, low mood, and feelings of boredom. This was captured by Evelyn who stated: “*The start of [the] COVID-19 [pandemic] was really stressful. I had flashbacks of being [isolated during cancer treatment]. Everyday there is a reminder of [the] COVID-19 [pandemic] […]. I am constantly stressed, constantly anxious […]. [The COVID-19 pandemic] has been a real rough ride. I have dealt with a lot of depression and suicidal thoughts […]*”. For many, lowered mental health was described as relating directly to the uncertainty of the pandemic, and fears of catching COVID-19. This was captured by John, who said: “*[Uncertainty during the COVID-19 pandemic] creates some anxiety. Because it’s hard to both mentally and physically know exactly what to do with each new phase [of the COVID-19 pandemic] that we’re passing through […]*”.

Others, however, shared ongoing challenges that simply co-occurred with the pandemic. As examples, some participants described pre-existing mental illness or physical conditions (in addition to cancer), their competing roles and responsibilities, and financial difficulties which were exacerbated by the COVID-19 pandemic. For example, Olivia elaborated on challenges due to pre-existing physical conditions when stating: “*I was turned away from the hospital three times [while I was] in cardiac arrest [because of the COVID-19 pandemic] […]. [I am] scattered because of the stress and anxiety […]. I am hyper-tuned into everything. My fears my stress, everything has been impacted by [the] COVID-19 [pandemic] and cancer […]. [I have been feeling] alone, helpless, frustrated, angry, scared. There’s so many emotions and it affects me physically and mentally*”.

#### 3.1.3. Social Isolation and Connection

Participants also described grappling with feelings of social isolation that were either due to public health restrictions or their own decisions to mitigate their likelihood of being exposed to COVID-19. Isabella shared: “*I used to do groceries with my neighbor [who is] really helpful and wonderful […]. With [the] COVID-19 [pandemic], we stopped doing that because I didn’t feel safe being in a car with them […]. Now, I get really lonely. I have no friends. I’ve been really stressed […]. And, you know, I have nobody to talk to […]. I wish I had more social support [to help with my stress]*”.

While some participants described feeling disconnected and isolated from their family, friends, and society in general during the COVID-19 pandemic, others were able to maintain or access social support. This was described as beneficial, and participants explained how their social network acted as a buffer to help them cope with the negative impacts of the pandemic and protect their mental health. This was captured by Sanja, who said: “*I think my friendships have deepened over the pandemic because we realized how much we need each other. [We are] supporting each other in really different ways, like bringing each other food, sharing food, finding ways to connect, being able just to go for walks, outside and stuff*”.

### 3.2. Main Findings

#### 3.2.1. Access to Supportive Care Programming: A Double-Edged Sword

When asked about supportive care programming, approximately half the sample indicated they had been told about supportive care programming after their cancer diagnosis (*n* = 60; 53.1%) and that they had accessed some form of supportive care programming (*n* = 55; 48.7%) since their diagnosis. When reflecting on their supportive care programming use in relation to the COVID-19 pandemic, 41.6% (*n* = 47) of participants indicated they had participated in some form of supportive care programming since the start of the pandemic. Of these 47 participants, 15 (31.9%) perceived their use to be greater, 16 (34.0%) perceived their use to be about the same, and 5 (10.7%) perceived declines in their use of supportive care programming (when compared to pre-pandemic).

During interviews, participants shared the varied types of supportive care programming they accessed through the pandemic (e.g., individualized coaching or counseling calls, support groups, physical activity programming). Regardless of the types of programming accessed, participants reflected on the marked changes in delivery through the COVID-19 pandemic, which impacted accessibility and led to new challenges. 

##### Remote Delivery Enhanced Access

For some participants, the transition to online delivery ensured their access to the same or similar supportive care programming as prior to the pandemic. For example, Leah said: “*I was told that I could join a fitness group for cancer [before COVID-19], but as the COVID [-19 pandemic] got more entrenched, they canceled it […]. Then, my oncologist told me about [an online physical activity] program […]. So, for 12 weeks, I did fitness for two days a week [virtually]. It gave me my life back, for sure*”. For other participants online programming increased their access beyond what they had prior to the COVID-19 pandemic. For example, Avery commented “*Because we’re in a small community, we often don’t get a chance to participate in stuff […]. [Online delivery] is handy for programs [and research] like this. I at least get to participate. I’m not driving to the city to do [a program or interview] like this*”. Indeed, for those residing in rural and remote locations, a sense of gratitude was shared as participants recounted the ways in which online delivery removed barriers.

##### Remotely Delivered Physical Activity Was Not without Its Challenges

Notably, for those accessing group-based physical activity (a common type of supportive care programming), the transition online was not as smooth. Some recounted fully cancelled programming and others shared that they still faced barriers to attending online options due to their competing demands and responsibilities. For example, Olivia shared: “*[Early in the COVID-19 pandemic], I participated in [physical activity programs] online. But now that I’m back to work, I’m not able to take part because it’s during the day*”. Other participants felt apprehensive about engaging in physical activity online due to fear of injury or lack of access to required equipment. For example, Natalia said: “*It’s hard for people with cancer [to engage in physical activity] if you don’t have equipment. One-on-one [training] is a lot better because they can see if you’re actually doing it correctly, or that you won’t get injured. It’s frustrating and scary, like you don’t want to get hurt*”. 

#### 3.2.2. Gathering through COVID-19: There Is No “One Size Fits All” Solution

At the time of the study, most participants (56.6%) indicated they were somewhat hesitant about returning to in-person supportive care programming in an indoor environment, with 28.3% (*n* = 32) reporting they were ‘not at all comfortable’ and 28.3% (*n* = 32) indicating there were ‘a little comfortable’. When asked about comfort gathering, just over half of the sample reported they would be comfortable returning to in-person supportive care programming if they were to meet 1:1 either outdoors (*n* = 71; 62.8%), indoors (*n* = 58; 51.3%), or in small groups (≤10 people) outdoors (*n* = 57; 50.4%). Of lower preference was gathering in small groups (≤10 people) indoors (*n* = 40; 35.4%), medium groups (10–20 people) outdoors (*n* = 24; 21.2%), medium groups indoors (*n* = 14; 12.4%), large groups (≥20) outdoors (*n* = 12; 10.6%), and large groups indoors (*n* = 4; 3.5%).

When asked about their primary concerns returning to in-person supportive care programming, most participants were concerned about the risk of catching or spreading COVID-19 (*n* = 72; 63.7%), being around others (*n* = 56; 49.6%), personal health status (*n* = 43; 38.1%), and uncertainty with the cleanliness of the environment (*n* = 30; 26.5%). Other concerns returning to in-person supportive care programming included transportation to programming (*n* = 14; 12.4%), leaving home (*n* = 9; 8%), or traveling to another community (*n* = 18; 15.9%). Of note, 15 (13.3%) participants had no concerns returning to in-person supportive care programming. 

When reflecting on their comfort gathering in-person, interview participants shared a range of sentiments. This included a desire to balance their need for connection with their own health and safety and their increased concerns about public health restrictions lifting. 

##### Balancing the Need for Connection and Comfort 

Most participants shared they wanted to return to in-person programming, but that they were nervous to do so. Participants described uncertainty regarding safety and expressed fear when considering gathering indoors with larger groups. This was captured by Jake, who shared: “*I want to do things, and just go out and meet new friends and enjoy family […] but I try to avoid people as much as possible, which is quite stressful [since I’m immunocompromised] […]. You just don’t know how safe it will be out there […]. [Even though] I’d want to meet new friends and do more activities*”. Participants described taking several precautions such as limiting interactions to only close family and friends, wearing a mask, and physically distancing, and were often reassessing their risk in various situations. As John shared: “*We went from not gathering at all [at the beginning of the COVID-19 pandemic], to gathering with people we know really well. As cases increase and more people go to the hospital, I will probably start to reduce the number of people I’m gathering with again […]. We are meeting outdoors [now that we are] coming into the summer […]. Every time it feels as though [the] COVID-19 [pandemic] goes down, we start to explore rejoining with our friends. And then, pull back when things seem to deteriorate*”. For many, the balancing act (i.e., concerns and risks of gathering and rewards of connecting) felt risky, which was captured when Alvin said: “*When you don’t know how it [COVID-19] will affect you, that feels like Russian roulette to me*”.

##### Disappointment and Frustration in the Face of Easing Public Health Restrictions and Violations 

Participants expressed their concerns when discussing lifting public health restrictions and when others did not follow rules. This was captured when Sanja said: “*It makes it a little bit riskier for me to even go to the grocery stores [with lifting restrictions]. A lot of people aren’t wearing masks anymore and there are no isolation requirements […]. So, it makes [life] a little bit riskier and adds an extra layer of complexity to navigate errands*”. Participants expressed a sense of frustration and hurt when people did not adhere to public health restrictions. They shared that as someone with a pre-existing medical condition for whom COVID-19 could be more severe, it was disheartening to see the lack of empathy and understanding from others. This was captured by Jake, who said: “*It’s stressful for me to see all the people on the streets with no mandate. They have no sympathy for those who are immunocompromised*”. Participants desired (possibly) longer and stricter enforcement of public health restrictions, which they felt were important for their own safety. 

### 3.3. Practical Suggestions for Moving Forward in the Context of COVID-19 

Participants endorsed several safety protocols, which they deemed important to enhance their comfort gathering in-person, whether indoors or outdoors. These included: physical distancing (indoors *n* = 78; 69%, outdoors *n* = 75; 66.4%), proof of vaccination (indoors *n* = 90; 79.6%, outdoors *n* = 74; 65.5%), hand sanitizing stations (indoors *n* = 70; 61.9%, outdoors *n* = 54; 47.8%), face masks and other personal protective equipment (indoors *n* = 82; 77%, outdoors *n* = 70; 61.9%), limiting attendees (indoors *n* = 76; 67.3%, outdoors *n* = 66; 58.4%), and proper airflow and ventilation (indoors *n* = 62; 54.9%, outdoors N/A). Temperature checks (indoors *n* = 24; 21.2%, outdoors *n* = 15; 13.3%), environments that are closed to the public (indoors *n* = 42; 37.2%, outdoors *n* = 41; 36.3%), and only being around people they knew (indoors *n* = 35; 31%, outdoors *n* = 32; 28.3%) were also endorsed by some. 

## 4. Discussion

This study described supportive care programming access and comfort gathering in-person. Less than half of the sample had accessed supportive care programming since their diagnosis and the onset of the COVID-19 pandemic. Of those who had accessed supportive care programming during the pandemic, similar or greater access was reported. With regards to comfort gathering for in-person supportive care programming, most participants in this sample expressed hesitation and desired programming that balanced access (i.e., hybrid, outdoor), their need for social connection, and safety. 

Most participants in this sample had not accessed supportive care programming, which may be because they are accessing resources to manage their disease elsewhere or may highlight a gap in care. As this is among the first studies to report on supportive care programming access (across all types of supportive care), further work is required to better understand who does and does not hear about and subsequently access supportive care programming. For those who had accessed supportive care programming since the start of the pandemic, online delivery was generally viewed favorably. Findings from this study therefore reinforce prior published research suggesting online supportive care programming may be a viable approach to address supportive care programming needs while overcoming common (e.g., cost, time, access; [23]) and COVID-19-related barriers (e.g., minimizing exposure to COVID-19; [24]). 

Nevertheless, supportive care programming was not free from challenges. Participants in this sample described feeling hesitant to engage in physical activity programming delivered online due to lack of equipment and concerns about safety. These findings support perspectives shared in a commentary at the onset of the COVID-19 pandemic [25]. Further efforts will be required to communicate that physical activity delivered online can be safe (when supervised by appropriate personnel) and that various household items (or cost-effective exercise bands) can be used as equipment. 

This is among one of the first studies to explore comfort gathering in-person among adults affected by cancer through the COVID-19 pandemic. Participants shared mixed views with regards to comfort gathering and concerns about COVID-19 that suggest it may be challenging to meet everyone’s needs in relation to both programming directives and safety. These findings speak to the highly political climate at the time of data collection and the polarizing views about restrictions related to COVID-19, which may have impacts that extend well-beyond the pandemic. 

Collectively, findings from this study inform four recommendations for those developing, refining, or offering supportive care programming through the COVID-19 pandemic that align with the American College of Clinical Oncology’s Recovery Report [26]. First, ensure online, hybrid, and outdoor options (weather permitting) are available. Second, in the context of physical activity programming, consider additional educational components for participants to address concerns for safety and lack of equipment, and be sure to use available videoconference features to promote opportunities for social connection. Third, develop detailed contingency plans in the case of future lockdowns/restrictions. Fourth, include a range of safety measures to enhance participant comfort and decrease the risk of spreading COVID-19. 

Although providing some insights into supportive care programming access and comfort gathering in-person through the pandemic, findings from this study should be interpreted while considering the following limitations. First, the survey and interview data were collected during two different phases of the COVID-19 pandemic. There may have been variations in mental health, perceptions of access, and perspectives of comfort gathering among adults affected by cancer between the two time points. As the COVID-19 pandemic evolves, program developers need to continue to adapt to address the changing needs of adults affected by cancer. Second, as this is a relatively novel area of research, there were no validated survey tools to explore supportive care programming use and comfort gathering in-person. Thus, the researchers generated the items comprising the questionnaires. Third, detailed information covering the types of supportive care programming accessed by participants in this sample was not collected. Looking ahead, gathering data covering the type(s) of supportive care programming accessed will be important to provide context that will be critical to better interpret findings. Fourth, the survey sample represents a very small proportion of adults affected by cancer who reside in Canada. Findings should not be generalized broadly and should be interpreted with this in mind. Similarly, participants self-selected to participate and could represent a biased sample of adults affected by cancer who may be more likely to advocate for and/or access supportive care programming. 

Findings from this study suggest online delivery may increase access to supportive care programming for some adults affected by cancer. There were mixed perspectives regarding comfort gathering in-person. This study provides evidence that may support the refinement and development of supportive care programming for adults affected by cancer through the COVID-19 pandemic.

## Figures and Tables

**Table 1:** Characteristics of survey and interview participants.

Variable	Survey Sample Mean (SD) *n* (%)	Interview Sample Mean (SD) *n* (%)
Age ^a^	61.9 (12.7), range = 21.0–84.0	58.0 (14.5), range = 35.0–77.0
Location (region) ^b^		
The West Coast	41 (36.3)	4 (33.3)
The Prairie Provinces	20 (17.7)	6 (50.0)
Central Canada	45 (39.8)	2 (16.7)
The Atlantic Provinces	6 (5.3)	00 (0)
The Northern Territories	1 (0.9)	00 (0)
Setting ^b^		
Urban (>100,000 people)	75 (66.4)	10 (83.3)
Rural (<99,999 people)	37 (32.7)	02 (16.7)
Biological sex ^b^		
Female	77 (68.1)	07 (58.3)
Male	35 (31.0)	04 (33.3)
Prefer not to answer	01 (0.9)	01 (8.3)
Education (highest level attained) ^b^		
Some/completed high school	10 (8.8)	2 (16.7)
Some university/college	25 (22.1)	02 (16.7)
Completed university/college	49 (43.4)	04 (33.3)
Some graduate school	07 (6.2)	02 (16.7)
Completed graduate school	22 (19.5)	02 (16.7)
Annual income (CAD) ^b^		
<$20,000	02 (1.8)	01(8.3)
$20,000–$39,000	18 (15.9)	02 (16.7)
$40,000–$59,000	19 (16.8)	04 (33.3)
$60,000–$79,000	19 (16.8)	02 (16.7)
$80,000 = $99,000	20 (17.7)	01 (8.3)
>$100,000	31 (27.4)	02 (16.7)
Ethnic origin ^‡,b^		
British	65 (57.5)	08 (66.7)
Western European	33 (29.2)	02 (16.7)
Eastern European	20 (17.7)	02 (16.7)
Northern European	07 (6.2)	02 (16.7)
Southern European	04 (3.5)	00 (0)
Aboriginal	05 (4.4)	02 (16.7)
East and Southern Asia	01 (0.9)	01 (8.3)
Caribbean	01 (0.9)	01 (8.3)
African	01 (0.9)	00 (0)
Other	10 (8.8)	2 (16.7)
Time since diagnosis (months) ^a^	56.8 (69.8), range = 1–368	53.8 (62.9), range = 4–219
Diagnosis ^‡,b^		
Blood	22 (19.5)	4 (33.3)
Breast	30 (26.5)	00 (0)
Digestive	07 (6.2)	02 (16.7)
Genitourinary	30 (26.5)	02 (16.7)
Gynecological	07 (6.2)	00 (0)
Head and neck	02 (1.8)	00 (0)
Lung	07 (6.2)	02 (16.7)
Neurological	02 (1.8)	00 (0)
Skin	06 (5.3)	00 (0)
Thyroid	04 (3.5)	00 (0)
Other	07 (6.2)	03 (25.0)
Treatment status ^b^		
Pre-treatment	05 (4.4)	00 (0)
On-treatment	36 (31.9)	03 (25.0)
Off-treatment	32 (28.3)	04 (33.3)
Palliative care	09 (8.0)	00 (0)
Time since treatment date (months) ^a^	52.4 (71.6), range = 2–356	54.4 (62.0), range = 10–187
Immune system status ^b^		
Physician-confirmed immunocompromised	48 (42.5)	06 (50.0)
Self-reported immunocompromised	68 (60.2)	09 (75.0)
Perceived Stress Scale Score ^†,a^ Proporation within each category ^b^	16.9 (8.2)	22.4 (12.0)
Low (0–13)	39 (34.5)	03 (25.0)
Moderate (14–26)	58 (51.3)	04 (33.3)
High (27–40)	16 (14.2)	05 (41.7)
UCLA Loneliness Scale score ^§,a^ Proporation within each category ^|,b^	17.5 (5.4)	21.4 (6.6)
Low (8–13)	28 (24.8)	03 (25.0)
Normal to moderate (14–20)	50 (44.2)	03 (25.0)
Moderate to high (20–25)	33 (29.2)	02 (16.7)
High (26–32)	09 (8.0)	04 (33.3)

Notes. ^a^ Mean (standard deviation; SD), ^b^ *n* (%), ^‡^ Participants could select ‘all that apply’, ^†^ Scale range = 0–4; score range = 0–40; higher scores indicate greater stress, ^§^ Scale range = 1–4, score range = 8–32; higher scores indicate greater loneliness. ^|^ There is overlap in score ranges for each of the categories, and thus, *n* ≠ 113. CAD = Canadian dollars.

**Table 2:** Theme descriptions and representative quotes from purposefully recruited study participants.

Theme	Subtheme(s)	Representative Quotes
The Context		
Mental Health Through COVID-19	—	“*Generally, I think I’ve been doing pretty good [through the COVID-19 pandemic]*”. Sanja “*There have been significant challenges. My wife still works, which has created a fair amount of stress […]. [The risk of bringing COVID-19 home] has created tension between the two of us and a fair amount of anxiety in me. And probably in her too because she’s worried about me*”. Alvin “*[The COVID-19 pandemic] has been a real rough ride. I have dealt with a lot of depression and suicidal thoughts […]. My concerns with my [pre-existing] Fibromyalgia and bipolar [disorder] lifted my level of anxiety […]*”. Evelyn “*[During the COVID-19 pandemic my] stress [has been] right up there. [I’ve been] really stressed […]. It’s just that at my age, and my lung situation, I have always felt that if I were to get COVID[-19] I’d probably die […]. So, yeah. I do feel nervous about [the] COVID[-19 pandemic]*”. Isabella
Social Isolation and Connection	—	“*My partner is very supportive. We share the house and support each other. [My partner] is much more social than I am. They keep me in contact with other people. My child has also moved home for this period. So, I have the family as a support here*”. John “*I do not have a support system at home […] [It has] been really isolating. Like during chemo treatment, I had to make special arrangements to be a group of one [in my academic classes] because I was immunocompromised and couldn’t go near other people in my program, so there was no real interaction [with others]*”. Natalia “*[I had] zero [support during the COVID-19 pandemic]. I lost friends, family, and people close to me because they were uncomfortable [with my diagnosis]. Some don’t want to get me sick [with COVID-19] and some don’t want to take precautions*”. Oliva “*[Being supported] has been pretty good. I’ve been blessed with a network of friends […]. We support each other*”. Evelyn
**Main Findings**		
Access to Supportive Care Programming: A Double-Edged Sword	Remote Delivery Enhanced Access	“*I attend many [remote supportive care programs] now. I spend the majority of my time online accessing everything from professional groups to yoga groups to exercise groups, and I really liked it […]*”. Kalvin “*I’m quite happy with doing online [programs] I’m not keen on going to a hospital […]. Getting [to the program in-person] by public transit takes the whole day. So, I’ve quite enjoyed doing stuff online*”. Isabella “*I did it [accessed supportive care programming] throughout [the COVID-19 pandemic] […]. It was good to have an online program. It was convenient*”. Natalia
	Remotely Delivered Physical Activity was not Without its Challenges	“*I find with live yoga over Zoom, that I feel like there’s too much pressure to do the poses how they do them on the screen. I can’t, or sometimes I just don’t want to extend [my body in that pose] […]. Maybe I’m an outlier. But for me, the live [online] classes never really hit the mark […]*”. Sanja “*It’s always hard to get started [in online programs]. I probably wouldn’t [participate in] an exercise group through Zoom. [I would prefer to] use the gym and establish a program for myself*”. John “*I couldn’t really connect with other people [because it was online] […]. They would talk about the importance of connections [in the program], but they didn’t use any of the tools that are available on Zoom […]. I couldn’t really connect with other people*”. Natalia “*I [participated] in a couple of [online yoga] classes during [the] COVID [pandemic] […] but it didn’t work very well because I wasn’t set up properly here. It was online so I couldn’t really see properly. I stopped [attending] […]. I miss that camaraderie [that I felt in-person]*”. Isabella
Gathering Through COVID-19: There is no “One Size Fits All” Solution	Balancing the Need for Connection and Comfort	“*I really want to gather and see friends. But I’m very concerned about large gatherings*”. Sanja“*Yeah. I’m pretty comfortable [gathering] outside [compared to gathering inside]. I’m pretty comfortable inside with a mask. When [gathering] inside without a mask, with people that I don’t know, then I’m less comfortable*”. Natalia “*[My family] and I have kept our bubble small due to [the fear of] COVID-19. Increasing our bubble] is a matter of getting used to things and slowly getting back to our community. Right now, our social life is at the bank, hardware store, or grocery store […]. I don’t even go to church […] and [going to church] was always something important to me, and I don’t feel safe now to do that. So, we haven’t gone to church in two plus years*”. Avery
	Disappointment and Frustration in the Face of Protocol Violations	“*I just get mad at the people that don’t believe it […]. I think mentally it’s just the frustration of […] the people that think it’s all a hoax […] and think they can go out and pull people’s masks off*”. Elise “*When [the government] dropped all the mask requirements, I got extremely uncomfortable […]. I like to say, ‘your freedom is my prison’. But nobody talks about those trade-offs*”. Alvin “*Where I live [people are] being very ignorant. They refuse to wear masks […]. People don’t want the mandates, unless they’re sick like me […]. It’s frustrating […]. Just because mandates are lifted, doesn’t mean hospitals, patients, and people aren’t still affected*”. Olivia

**Table 3:** Additional details for quotes of interview participants.

Pseudonym	Age	Sex	Cancer Type	Treatment Status
Elise	68	Female	Colorectal cancer	Not reported
Sanja	35	Female	Colorectal cancer	On-treatment
Leah	77	Female	Lung cancer	On-treatment
Jake	41	Male	Sarcoma	Off-treatment
John	72	Male	Prostate cancer	Off-treatment
Natalia	47	Female	Hodgkin’s lymphoma	Off-treatment
Alvin	52	Male	Non-Hodgkin’s lymphoma	Off-treatment
Avery	56	Female	Hodgkin’s lymphoma	Off-treatment
Kalvin	68	Male	Prostate cancer	On-treatment
Isabella	74	Female	Lung cancer	Pre-treatment
Oliva	48	Female	Other ^a^	Not reported
Evelyn	Not reported	Female	Non-Hodgkin’s lymphoma	Off-treatment

Note. ^a^ Participant selected ‘other’ and during the interview specified they had AL cardiac amyloidosis, which is associated with blood cancers. In discussions with study team members, the decision was made to retain the participant in analyses.

## Data Availability

The data cannot be shared as participants were assured that their data would be kept private and confidential and that only the study team would have access to their raw data. However, summaries of the de-identified data are available upon reasonable request to the corresponding author.

## Supplementary Materials

The following supporting information can be downloaded at: https://www.mdpi.com/article/10.3390/curroncol30030198/s1, Supplementary File S1: Closed- and open-ended researcher-generated items assessing supportive care programming access and comfort gathering.
