# Supplementary material for: Describing Supportive Care Programming Access and Comfort Gathering through the COVID-19 Pandemic: An Observational Mixed Methods Study with Adults Affected by Cancer
Source: Curr Oncol. 2023 Feb 22;30(3):2598–612. doi: 10.3390/curroncol30030198 (PMC10047440; doi:10.3390/curroncol30030198)
Supplement: Supplementary file 1 [file curroncol-30-00198-s001.zip › curroncol-2207368-supplementary.pdf]

Supplementary File S1
Closed- and open-ended researcher-generated items assessing supportive care programming access and comfort gathering

1. Were you told about supportive care programming (i.e., free or fee-based programming to address the psychological, emotional, social, spiritual and physical concerns of cancer) after your cancer diagnosis?

☐Yes
☐No

2. Have you ever engaged in supportive care programming related to your cancer diagnosis?

☐Yes
☐No

3. Since the start of the pandemic, did you participate in supportive care programming?

☐Yes
☐No

i) If yes, did you participate in more or less supportive care programming than before COVID-19?
☐Much more
☐More
☐About the same
☐Less
☐Much less

4. Please use this space for any other comments about your engagement in supportive care programming during COVID-19.

5. What level of COVID-19 precautions are you currently practicing?
☐Primarily self-isolating
☐Mostly self-isolating at home but going into the community for essentials or appointments
☐Socializing in small groups, occasionally with physical distancing
☐Not taking any precautions at all
☐Other, please specify:

6. What are your primary concerns about starting or returning to <u>IN-PERSON</u> supportive care and/or physical activity programming? Please check all that apply.
☐Transportation
☐Being around others

☐Leaving home
☐Travelling to another community
☐Personal health status
☐Risk of catching or spreading COVID-19
☐Uncertainty with cleanliness of the facility or environment
☐No childcare support
☐I have no concerns
☐Other, please specify:

7. Which environment(s), if any, are you comfortable with outside of your home? Please check all that apply.
☐Not comfortable outside my home yet
☐One on one meeting outdoors
☐One on one meeting indoors
☐Small group (10 people or less) event outdoors
☐Small group (10 people or less) event indoors (cafe, restaurant, office etc.)
☐Medium group (10-20 people) event outdoors
☐Medium group (10-20 people) event indoors (cafe, restaurant, community centre etc.)
☐Large group (20+ people) event outdoors
☐Large group (20+ people) event indoors (community centre, restaurant, hall etc.)
☐Other, please specify:

8. How comfortable would you be participating in programming IN-PERSON, INDOORS right now?
☐Not at all comfortable
☐A little comfortable
☐Somewhat comfortable
☐Quite a lot comfortable
☐Completely comfortable

9. What general conditions or safety protocols would have to be in place for you to feel comfortable attending supportive care programming IN-PERSON, INDOORS? Select all that apply.
☐2 meter distancing
☐Vaccine passports
☐Outdoors only
☐Temperature checks
☐Hand sanitizing station
☐Face masks and other personal protective equipment
☐Limited number of attendees
☐Environment that is not open to the general public

☐Good airflow
☐Being around people I know
☐I am not comfortable with in-person, indoor gathering

10. How comfortable would you be participating in programming IN-PERSON, OUTDOORS right now?
☐Not at all comfortable
☐A little comfortable
☐Somewhat comfortable
☐Quite a lot comfortable
☐Completely comfortable

11. What general conditions or safety protocols would have to be in place for you to feel comfortable attending supportive care programming IN-PERSON, OUTDOORS? Select all that apply.
☐2 meter distancing
☐Vaccine passports
☐Outdoors only
☐Temperature checks
☐Hand sanitizing station
☐Face masks and other personal protective equipment
☐Limited number of attendees
☐Environment that is not open to the general public
☐Being around people I know
☐I am not comfortable with in-person, outdoor gathering
